# An Unusual Case of Hemolytic Anemia: A Case Report

**DOI:** 10.7759/cureus.75676

**Published:** 2024-12-13

**Authors:** Ana Patrícia Moreira, Mafalda Vasconcelos, Teresa Costa e Silva, Catarina Relvas, João Espírito Santo

**Affiliations:** 1 Internal Medicine, Hospital Beatriz Ângelo, Lisboa, PRT; 2 Oncology, Instituto Português de Oncologia de Lisboa Francisco Gentil, Lisboa, PRT

**Keywords:** hemolytic anemia, megaloblastic anemia, pancytopenia, pseudo-thrombotic microangiopathy, vitamin b12 deficiency

## Abstract

Vitamin B12 deficiency is a potentially severe condition with clinical manifestations ranging from nonspecific symptoms, such as asthenia and glossitis, to severe hematological problems, including pancytopenia and megaloblastic anemia. One of the rare phenomena associated with this condition is pseudo-thrombotic microangiopathy (pseudo-TMA), which can mimic diseases such as thrombotic thrombocytopenic purpura (TTP), leading to possible misdiagnosis and inappropriate treatment.

In this article, we present the case of a 62-year-old man with a history of intravenous drug use, untreated hepatitis C, smoking, and alcoholism. The patient was admitted to the emergency department with progressive asthenia, fever, and disorientation. Laboratory findings revealed severe pancytopenia, schistocytes on the peripheral blood smear, and elevated lactate dehydrogenase levels, suggesting a microangiopathic condition. After investigation, a severe deficiency of vitamin B12 and folate was identified, and the condition was diagnosed as pseudo-TMA secondary to this deficiency.

The treatment consisted of intramuscular vitamin B12 administration, resulting in the gradual normalization of the blood count and resolution of symptoms. This case highlights the importance of considering vitamin B12 deficiency in the differential diagnosis of patients with hemolytic anemia and thrombocytopenia, avoiding unnecessary interventions, such as plasmapheresis, and allowing for a quick and effective recovery with appropriate vitamin replacement.

## Introduction

Vitamin B12 (cobalamin) is essential for deoxyribonucleic acid (DNA) synthesis and the proper formation of red blood cells. Its deficiency leads to defective DNA synthesis in the bone marrow, resulting in the production of large, immature red blood cells that are often destroyed within the bone marrow itself (intramedullary hemolysis) before being released into the bloodstream.

The typical presentation of B12 deficiency (below 200 pg/mL or 148 pmol/L) is megaloblastic anemia or pancytopenia in severe cases, while other presentations are rare. Symptoms range from fatigue, glossitis, and subtle neurological impairment in mild to moderate cases, to severe hematological problems, neurological manifestations, and cardiomyopathy in more severe cases [1].

Pseudo-thrombotic microangiopathy (pseudo-TMA) is a condition that mimics thrombotic microangiopathies, such as thrombotic thrombocytopenic purpura (TTP) and hemolytic-uremic syndrome, but has distinct underlying causes. These include genetic factors (such as congenital or acquired ADAMTS13 deficiency), infections, malignancies, autoimmune diseases, pregnancy, or adverse reactions to specific medications. Severe vitamin B12 deficiency (below 100 pg/mL or 74 pmol/L) can present as pseudo-TMA in approximately 2.5% of patients [2]. Similar to TTP, there is red blood cell destruction with the presence of schistocytes on the blood smear, thrombocytopenia, and symptoms that may include neurological manifestations (such as confusion and disorientation), as well as fatigue, pallor, and jaundice.

Due to its clinical similarity to true thrombotic microangiopathies, pseudo-TMA secondary to vitamin B12 deficiency can delay diagnosis or lead to misdiagnosis, putting the patient at significant risk. Timely recognition and treatment of vitamin B12 deficiency are crucial to avoid unnecessary and potentially harmful interventions [1], as demonstrated by the patient’s rapid improvement following supplementation. Given the low cost and ready availability of testing, a recommendation to evaluate all patients presenting with possible thrombotic microangiopathy for vitamin B12 deficiency could be considered.

## Case presentation

We describe the case of a 62-year-old Caucasian man who presented to the emergency department of our hospital with a 20-day history of progressive asthenia.

The patient had a prior history of intravenous drug use, untreated hepatitis C, active smoking, and alcohol use disorder. He denied any previous surgeries, was not on regular medication, and had no known drug allergies.

Upon admission, he was febrile (tympanic temperature of 38ºC), disoriented in time, with atrophic glossitis and multiple missing teeth, scaly skin on the palms of the hands, and distal hair thinning on the lower limbs, without lymphadenopathy, organomegaly, or other notable physical examination findings.

**Figure 1:**
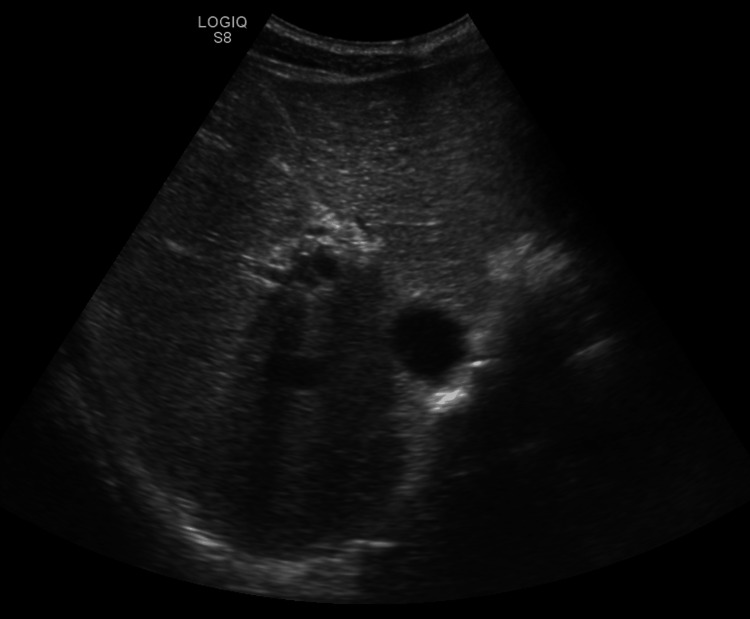
Abdominal ultrasound showing normal-sized liver with regular contours, showing mild steatosis and multiple infra- and juxta-centimetric cystic images scattered throughout both lobes.

Laboratory evaluation revealed severe pancytopenia (Table 1); peripheral blood smear with pancytopenia, marked anisopoikilocytosis and anisocytosis, many schistocytes, leukopenia without atypical cells, and platelet anisocytosis; HIV serology negative. Abdominal ultrasound documented signs of hepatic steatosis (Figure 1).

**Table 1:** Investigation profile of the patient at the time of admission.

Investigations	Patient	Reference values
Hemoglobin	3.5 g/dL	12-16 g/dL
Mean corpuscular volume	102 fL	80-100 fL
Total leukocyte count	1860/dL	4,000-10,000/dL
Platelet count	57000/dL	150,000-400,000/dL
Total bilirubin	3.13 mg/dL	<1.2 mg/dL
Lactate dehydrogenase (LDH)	>5000 U/L	135-214 U/L
Serum creatinine	0.68 mg/dL	0.5-1.2 mg/dL
C-reactive protein	1.2 mg/dL	<0.5 mg/dL

**Figure 2:**
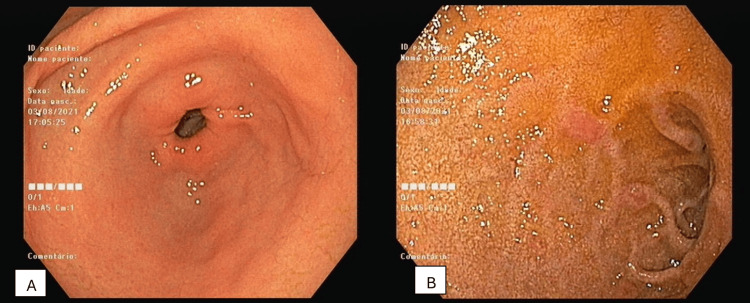
Upper endoscopy showing the gastric antrum (A) and bulb (B), where the mucosa appears papular and hyperemic, with no lesions

He was admitted for further evaluation of pancytopenia and received transfusional support with four units of packed red blood cells, resulting in an increase of hemoglobin to 7.1 g/dL. The study revealed severe deficiencies in vitamin B12 (143 ng/L) and folate (1.1 ng/mL); iron at 204 µg/dL, ferritin at 336 µg/L; haptoglobin <8 mg/dL; direct Coombs test negative. Endoscopy revealed generalized atrophic gastritis (Figure 2) and tests for anti-parietal cell, intrinsic factor and transglutaminase antibodies were negative.

No recurrence of fever was observed during hospitalization. When questioned, the patient reported a chronically inadequate diet (lacking fruits, vegetables, and very low in protein) associated with his alcohol use and socio-economic status.

Thus, a diagnosis of pseudo-TMA in the context of severe nutritional vitamin B12 deficiency was established. Intramuscular vitamin B12 replacement therapy was initiated (1mg injection), starting with daily administration, followed by weekly doses during the first month, and transitioning to monthly maintenance. This treatment led to the normalization of the blood count.

## Discussion

This case underscores the often-overlooked potential of severe vitamin B12 deficiency to present as pseudo-TMA, a rare but critical complication.

The diagnostic challenge in this case primarily lies in distinguishing pseudo-TMA from true thrombotic microangiopathies, such as TTP. TTP is characterized by the formation of thrombi in the arterioles and capillaries of various organs, leading to widespread platelet consumption and resulting in thrombocytopenia. These clots fragment red blood cells as they pass through narrowed vessels, causing microangiopathic hemolytic anemia. The obstruction of blood flow by these thrombi leads to organ damage, particularly affecting the brain, kidneys, and heart. If untreated, TTP has a high mortality rate, emphasizing the need for early diagnosis and immediate treatment, typically with plasmapheresis to remove the autoantibodies that inhibit ADAMTS13, the enzyme responsible for preventing excessive clot formation [3].

In this case, the critical point is the importance of vitamin B12 in DNA synthesis, the division and maturation of hematopoietic cells, and normal neurological function. Vitamin B12 acts as a cofactor in three key enzymatic processes: the conversion of 5-methyltetrahydrofolate to tetrahydrofolate, the conversion of methylmalonic acid to succinyl-coenzyme A, and the conversion of homocysteine to methionine. The accumulation of methylmalonic acid leads to progressive nerve damage (demyelination), which can cause neurological symptoms such as impaired proprioception, loss of vibratory sensation, peripheral neuropathy, and ataxia. The disruption of DNA synthesis due to the inability to produce tetrahydrofolate often presents as megaloblastic anemia, characterized by a mean corpuscular volume (MCV) greater than 100 fL [4-6]. However, it can also lead to more severe bone marrow failure, causing thrombocytopenia, neutropenia, and even pancytopenia. Elevated homocysteine levels can contribute to hemolysis by generating reactive oxygen species that damage cell membranes through lipid peroxidation, promoting endothelial injury, platelet aggregation, and coagulation activation [7].

In vitamin B12 deficiency, the bone marrow produces RBCs ineffectively, leading to intramedullary hemolysis, where immature RBCs are destroyed within the bone marrow. This process can mimic a thrombotic microangiopathy (TMA), with blood smear findings such as schistocytes, thrombocytopenia, and elevated LDH. Although the deficiency impairs DNA synthesis, it does not involve actual microvascular thrombi or endothelial vessel damage. In this case, the prompt identification of the cause of pancytopenia prevented unnecessary tests, such as bone marrow biopsy, and avoided unwarranted treatments, such as plasmapheresis, which carry inherent risks. Timely recognition and treatment of vitamin B12 deficiency are essential to prevent unnecessary and potentially harmful interventions [1]. Monitoring potassium levels during treatment is important due to the risk of hypokalemia in this context. The patient’s rapid improvement following the initiation of therapy highlights the critical importance of prompt diagnosis and management.

## Conclusions

This case underscores the importance of recognizing severe vitamin B12 deficiency as a potential cause of pseudo-TMA. Vitamin B12 deficiency disrupts DNA synthesis, leading to ineffective hematopoiesis and intramedullary hemolysis, which can mimic TMA by presenting with schistocytes, thrombocytopenia, and elevated lactate dehydrogenase levels on blood smears. Unlike true TMA, however, pseudo-TMA does not involve actual microvascular thrombosis or endothelial injury. 

This case highlights that early recognition and treatment of severe vitamin B12 deficiency can lead to significant clinical improvement, preventing unnecessary interventions. Clinicians should maintain a high index of suspicion for vitamin B12 deficiency in patients presenting with pancytopenia and microangiopathic findings, especially when accompanied by clinical signs like glossitis and a history suggestive of poor nutritional intake. Timely management can avert irreversible complications and improve patient outcomes, emphasizing the importance of thorough differential diagnosis in hematologic evaluations.
